# Salivary Antioxidant Capacity and Magnesium in Generalized Anxiety Disorder

**DOI:** 10.3390/metabo13010073

**Published:** 2023-01-02

**Authors:** Elena V. Proskurnina, Krystsina M. Liaukovich, Lyubov S. Bychkovskaya, Ivan V. Mikheev, Evgenia I. Alshanskaia, Mikhail A. Proskurnin, Olga V. Martynova, Galina V. Portnova

**Affiliations:** 1 Laboratory of Molecular Biology, Research Centre for Medical Genetics, 1 Moskvorechye St., 115522 Moscow, Russia; 2 Laboratory of Human Higher Nervous Activity, Institute of Higher Nervous Activity and Neurophysiology of the Russian Academy of Sciences, 5A Butlerova St., 117485 Moscow, Russia; 3 Department of Chemistry, Lomonosov Moscow State University, 1-3 Leninskie Gory, 119991 Moscow, Russia; 4 Faculty of Biology and Biotechnology, HSE University, 20 Myasnitskaya St., 101000 Moscow, Russia

**Keywords:** salivary antioxidants, salivary magnesium, generalized anxiety disorder, problem-solving model of anxiety

## Abstract

Generalized anxiety disorder (GAD) is a prevalent disorder. The search for biomarkers may contribute to new knowledge about molecular pathogenesis and treatment. Since oxidative stress and micronutrient imbalance play a key role in the development of mental disorders, we aimed to study salivary antioxidant capacity and magnesium in patients with GAD in an anxiety model of solving problems with increasing complexity. The study subgroup consisted of 15 patients with GAD, and 17 healthy volunteers of the same age made up the control subgroup. Participants took a test with six levels of difficulty, which included false feedback. In this test, the participants were asked to remember the colors of balloons and react when the color changed. The reaction time, the number of correct answers, as well as biochemical parameters such as the antioxidant capacity of saliva and salivary magnesium, were assessed. There was no difference in the results of the quest between the subgroups; however, anxious participants spent more time at the moment of experimental frustration due to incorrect feedback and additional negative psycho-emotional load. Antioxidant capacity did not differ between the subgroups both before and after the experimental session. Average antioxidant capacity also did not change significantly at the endpoint of the experiment. However, the endpoint antioxidant capacity correlated negatively with the reaction time in anxious patients in the second block (where the false feedback as a frustrating factor appeared). Magnesium was initially significantly higher in the group of anxious participants and decreased at the experiment endpoint; in healthy patients, there were no changes in salivary magnesium at the endpoint. In conclusion, the compensatory potential of oxidative metabolism and magnesium in patients with GAD was spent with additional psycho-emotional stress, in contrast to healthy individuals, but it was sufficient to avoid exhaustion during experimental frustrating exposure.

## 1. Introduction

Generalized anxiety disorder (GAD) is a mental disorder that is often poorly diagnosed and treated [1]. This illness is especially common among young people and is associated with other mental and somatic disorders [2]. GAD is diagnosed primarily based on symptoms and does not have specific laboratory tests because of a lack of knowledge on biomarkers. There are several studies on the morphology and biochemistry of GAD, but they are scattered and clearly not sufficient and have not yet been translated into clinical practice. To date, the main morphological biomarkers are obtained from neuroimaging studies, including various variants of magnetic resonance imaging (MRI), positron emission tomography/single-photon emission computed tomography, and proton magnetic resonance spectroscopic imaging methods [3,4,5]. As for the biochemical analysis, plasma is mostly used as it contains certain brain metabolites that penetrate the blood–brain barrier and can be quantified for proteomic and metabolomic analyses. There are only a few studies devoted to the investigation of molecular biomarkers in blood. Among them, measuring serotonin-related biomarkers have found to be decreased, in contrast to other anxiety disorders [6]. After a stressful test, there was no serious imbalance in the metabolism of the hypothalamus–pituitary–adrenal axis in male patients with GAD, and cortisol plasma levels did not change [7].

Challenge tests to provoke anxiety are another approach to study the biomarkers of GAD. Almost all works are aimed at studying cortisol and changing it during treatment, while the results are contradictory and do not allow a clear picture to be formulated. The administration of 7.5% CO_2_ did not significantly affect the cortisol in saliva in GAD patients [8]. As for neurotrophic factors, it seems that they are not significant in GAD, or information about them is contradictory [9]. Among inflammation biomarkers, several studies demonstrated an increase in C-reactive protein in GAD [10]. Interleukin 10 and α-melanocyte-stimulating hormone were increased in patients with GAD [11]. GAD was associated with an increased inflammatory index [12]. An interesting study was performed on the characteristics of the microbiome in GAD, which has diagnostic and therapeutic significance [13]. Overall, more studies on biochemistry are needed to provide a biological basis of pathogenesis and treatment of GAD.

The metabolism of reactive oxygen species (ROS) is basic for animals and humans. Low levels of ROS are necessary for physiological functioning, but in many pathologies, the balance shifts mainly towards an increase in ROS (oxidative stress). Studies of oxidative balance in GAD are extremely scarce. An increased lipid hydroperoxide level and decreased paraoxonase activity in serum were demonstrated elsewhere [14]. A decreased serum sulfhydryl level was observed in pure GAD patients, which indicates the redox disbalance [15]. The total antioxidant status and oxidative stress index in blood were assessed to prove oxidative stress in GAD pathophysiology [16]. Prolidase activity was increased in GAD patients [17]. Nitrosative stress and lipid oxidative stress along with lowered lipid antioxidants were demonstrated along with increased uric acid levels [18,19]. Another study revealed an increased level of malondialdehyde, and immunoglobulins, and decreased amounts of antioxidant vitamins A, C, and E [20].

Magnesium is an element that affects the functioning of the central nervous system. Stress depletes magnesium, and low micronutrient levels lead to stress [21]. Graphite furnace and flame atomic absorption spectroscopy were applied to quantify concentrations of trace elements (Zn, Cu, Mn, and Fe), Ca, and Mg in serum [22]. Serum Zn was significantly decreased while Cu, Mn, and Fe were significantly increased. The differences in the concentrations of Ca and Mg were not significant. On the other hand, there is evidence of the efficacy of a dietary supplement with magnesium on adjustment anxiety disorders [23].

Metabolomics studies unique chemical metabolites (metabolite profiles) in biological samples. Metabolite profiles are a kind of ‘bertillonage’ of physiological or pathological states that can provide reliable diagnostic information. Among other bioanalytical fluids, saliva is now recognized as an excellent diagnostic medium as it contains many informative metabolites [24]. Like blood, saliva contains many components such as proteins, antibodies, enzymes, mRNAs, DNAs, hormones, microelements, and other metabolites that are pathogenetically associated with the disease [25]. Since the collection of saliva is completely painless and non-traumatic, it does not cause stress, which is very important when studying the metabolism in mental illnesses such as anxiety disorder. As an analytical object, saliva has the advantage of not coagulating. Recent and potential developments of saliva in diagnostics in metabolomics are described in numerous reviews [24,26,27]. They emphasized that the saliva metabolome can be used not only in the diagnosis of diseases of the oral cavity, but also in systemic diseases. The saliva metabolome gives valuable information for the diagnosis of periodontal diseases [28], oral cancer [29,30,31], and cancer of the gastrointestinal tract [32].

A specific feature of saliva is the fact that it also reflects the local situation in the oral cavity. Due to salivary antioxidant substances, saliva plays a certain role in preventing and/or modulating ROS imbalance and inflammation in the oral cavity as it is, among others, a first line of defense against oxidative stress [33]. To date, several methods have been developed for assaying the antioxidant activity of saliva. However, when studying the antioxidant status of saliva, there are certain influencing factors regarding food intake, gender, smoking, oral hygiene, and oral pathologies [34]. Unstimulated saliva normalized by flow rate is preferable to measure the salivary antioxidant status [35]. The total antioxidant capacity is a reliable indicator of the antioxidant salivary potential correlating with the blood antioxidant potential, which depends primarily on the content of uric acid [36].

State-of-the-art methods for the analysis of salivary magnesium are presented in the review [37]. Inductively coupled plasma mass spectrometry is the most reported method for detecting ions in saliva; flame atomization atomic absorption spectroscopy is also frequently used. The concentrations of magnesium ions obtained in numerous studies differ quite strongly, in some cases by an order of magnitude. The authors attribute this to differences in the procedures for the collecting, storing, and sample preparation of saliva samples in different studies. Data on the concentration of magnesium in saliva in healthy individuals differ significantly (Table 1).

**Figure 1:**
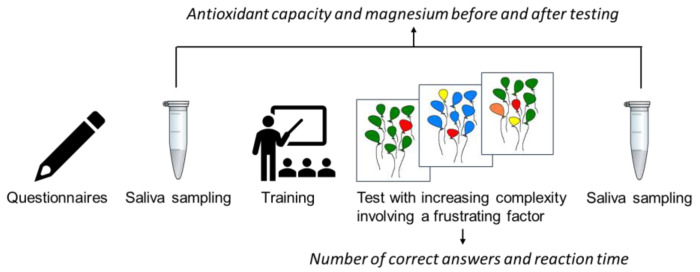
The general scheme of the experimental study.

In sum, the available studies on the study of biomarkers in GAD are fragmentary and few. Without a doubt, the list of relevant biomarkers can and should be expanded. New biological insights could help in improving diagnoses and developing new biomarker-based treatments of GAD. Here, we studied the antioxidant capacity of saliva and salivary magnesium in patients with GAD in a problem-solving model of anxiety. As far as we are concerned, to date, no such studies have been conducted. We have selected an anxiety model of problem solving with increasing complexity in GAD patients and healthy volunteers. Incorrect feedback has been used as a frustrating factor for an additional psycho-emotional load. Behavioral parameters and changes in the antioxidant potential of saliva and salivary magnesium were studied in comparison with the healthy control group (Figure 1).

## 2. Materials and Methods

### 2.1. Participants

In total, 17 healthy right-handed subjects and 15 right-handed patients with *generalized anxiety disorder* (GAD—F41.1 by International Statistical Classification of Diseases (ICD-10)) participated in our study (Table 2). The group of patients included only individuals who achieved remission longer than two months ago and did not apply any medications.

Exclusion criteria were as follows: aged younger than 20 and older than 50, menstrual cycle phase, oral contraceptive administration, previous neurological or other psychiatric history (other than F40–41), pregnancy, and treatment with antidepressants and anxiolytics on the moment of the study. No epileptic activity was registered in the electroencephalograms during the study. All participants had signed the informed consent for research document indicating their willingness to participate in the study. The study took place in the years of 2021–2022.

### 2.2. Questionnaires

**Figure 2:**
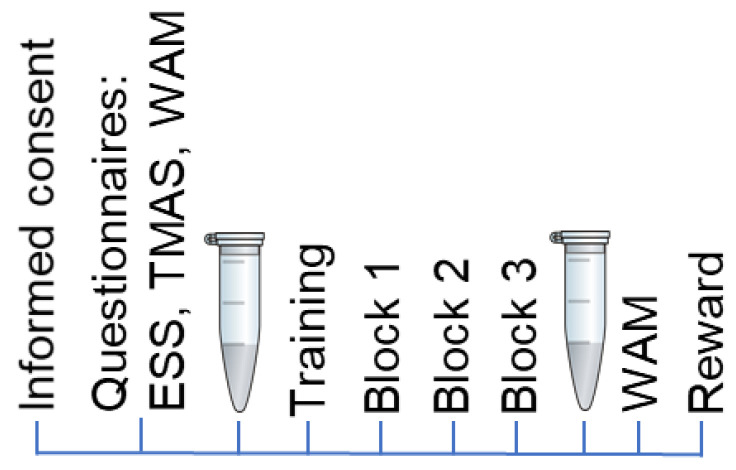
The schematic presentation of experimental steps; EES is the Epworth Sleepiness Scale; TMAS is Taylor’s Manifest Anxiety Scale; WAM is the ‘Wellbeing. Activity. Mood’ Questionnaire. The reward at the final stage of the test depended on the number of correct answers.

After signing the informed consent form, study participants completed the Epworth Sleepiness Scale (ESS) [45] to exclude people with excessive daytime sleepiness (higher than 13 points). Taylor’s Manifest Anxiety Scale (TMAS, 50 item version) [46] was used to measure the level of personal anxiety in both groups. The ‘Wellbeing. Activity. Mood’ Questionnaire (WAM) [47] is a Russian-developed questionnaire to assess the current state of a person. Before recording the main experimental series, the task was explained to the participants. Next, a practice training was held as many times as needed for the participant. Only when the task was understood completely, and all tests were performed correctly, did the main experimental session start. After the experimental session, the participants completed the WAM Questionnaire once more (Figure 2).

### 2.3. Stimuli and Protocol

The stimuli have previously been tested elsewhere [48,49,50].

The task had six levels of difficulty. Each participant was presented with pictures of balloons of assorted colors. Green and blue colors were the background ones. All other colors (pink, yellow, red, brown, orange, purple, and grey) were part of the task. The participants were to remember the colors of the balloons. If the color changed in the presentation of the next stimulus, they should press the ‘2’ button. If the color remained the same, the participants should press the ‘1’ button.

The difficulty level was determined by the number of balloons that changed color. At the first level of difficulty, only one balloon could change color. Thus, the participant had to memorize only one color. At the sixth level of difficulty, six colors had to be remembered.

The instruction for the participants was as follows: “Pictures with balloons of different colors will appear in front of you. You will need to compare the picture with the previous one and answer if the colors match. All colors are part of the game except blue and green. If you see all blue or all green balloons, you do not need to answer. Pictures will appear for a brief time. Try to answer as accurately and quickly as possible. If the colors match, press key ‘1’; if they differ, press key ‘2’. Balloons of the same colors are a signal that the level is over, and you will be asked to evaluate how well you coped with the task. In this game, the position of the balloons is not important, only the colors matter. If you answer correctly, a happy emoticon appears, if incorrectly or if you did not have time to answer within three seconds, a sad emoticon appears. At the end of the game, you will receive 250 rubles regardless. The more correct answers, the higher the winnings. The maximum additional win is 750 rubles”.

**Figure 3:**
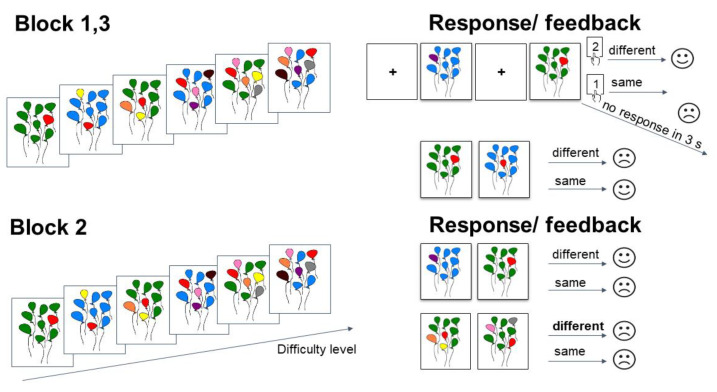
The block scheme of the experimental study. The ‘Different’ point is when colors differed; the ‘Same’ point is when colors did not differ; ‘s’ means seconds. In Blocks 1 and 3, the participants were given correct feedback. In Block 2, the participants were given wrong feedback at the 3rd level in some trials.

The main experimental series consisted of three blocks (first, second, and third block). Each block consisted of six difficulty levels. Each level consisted of 17 trials. In total, there were 102 trials in the first and the second blocks: 6 trials, in which the first balloon picture in each level had to be memorized but not reacted to; 49 trials in which the balloon picture did not match the previous one, requiring key ‘2’ to be pressed; 41 trials in which the balloon picture matched the previous one, requiring key ‘1’ to be pressed; and 6 trials in which a picture with either blue or green balloons at the end of each level signaled completion of the level, and no response was required. The second block differed from the first and the third blocks. The architecture was the same, but starting from the third level, participants were given the wrong feedback (a sad emoticon as if they made a mistake even though they were correct). In total, there were 20 trials with the wrong feedback. The duration of presentation of the feedback emoticon (size 20.3° × 13.6°) was 1 s. Each block was followed by a 30 s rest, during which the subject could remove his head from the chin rest and move. The block scheme of the experimental study is presented in Figure 3.

### 2.4. Behavioral Data

The times taken to complete each trial (the time from the appearance of the balloon picture to pressing a key to answer), each block, and each level were analyzed separately. The data were obtained using EyeLink Experiment Builder 2.3.1 (Mississauga, ON, Canada: SR Research Ltd., 2020). The reaction time (RT) and the numbers of correct and incorrect answers were counted after the completion of the experimental session, and the corresponding reward was given to the volunteer at the end of the experiment. The amount of winnings was calculated by the number of correct answers and was considered as one of the indicators of the success of solving problems.

### 2.5. Saliva Sampling

We analyzed non-stimulated saliva collected by spitting for 15 min before and after the experimental session. All tests and samplings were performed on an empty stomach from 10.00 a.m. to 13.00 a.m. The participants did not drink alcohol or caffeine-containing drinks from 20.00 p.m. on the day before the experiments. Two hours before the experiment, the participants brushed their teeth and did not drink any more after that. Before spitting, the participants pre-rinsed their mouths with cool still water. The volume of saliva collected was at least 1.5 mL. After centrifugation for 15 min at 2000× *g*, the samples were divided into two parts. A part of 600 µL was used for the assessment of antioxidant capacity during a day. The rest of the sample was frozen at −20 °C and used for magnesium quantitation within 1 month.

### 2.6. Assessment of Salivary Antioxidant Capacity

**Figure 4:**
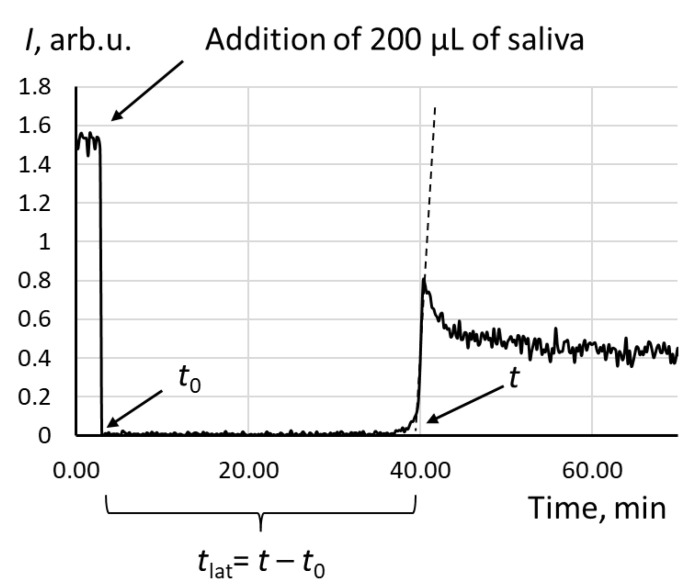
The chemiluminogram of a saliva sample of a healthy volunteer; the system contains ABAP (2.5 mM), luminol (2 µM), and saliva (200 μL); the latency time *t*_lat_ was used as an analytical signal.

A 12-channel Lum-1200 chemiluminometer with PowerGraph 3.3 Professional software (DISoft, Moscow, Russia) was used for the experiments. The salivary antioxidant capacity was assessed using a chemiluminescence protocol [51]. The chemiluminescent system consisted of 2,2′-azobis (2-amidinopropane) dihydrochloride (ABAP, Sigma-Aldrich, St. Louis, MO, USA) as a source of ROS and luminol (Sigma-Aldrich, St. Louis, MO, USA). The chemiluminescence reagent contained luminol (1 mmol/L) and ABAP (50 mmol/L) in a phosphate buffer solution (100 mM KH_2_PO_4_, pH 7.4, Sigma-Aldrich, St. Louis, MO, USA). After a cuvette with the chemiluminescence reagent was heated to 37 °C, the initial chemiluminescence was recorded and an aliquot of saliva (200 µL) was added. Chemiluminescence was recorded until the new stationary signal was achieved (Figure 4). The antioxidant potential was calculated as a latency time (*t*_lat_), which is the time from the addition of saliva to the intercept on the *x*-axis tangent to the area of maximum rise (Figure 4). Mean values of the antioxidant capacity (*t*_lat_) were calculated from triplicate data.

### 2.7. Quantitation of Magnesium

A magnesium standard solution with a concentration of 50 μg/L was prepared by diluting the standard with 1000 μg/mL (Inorganic Ventures, Christiansburg, VA, USA), 0.2% nitric acid obtained by diluting concentrated nitric acid, and 0.1% Triton X-100 (Merck, Darmstadt, Germany). Milli-Q deionized water was used to prepare the solutions (Millipore Corp., Darmstadt, Germany).

Immediately before the analysis, the samples were kept at room temperature (20–22 °C) until a temperature equilibrium was reached. Each sample was homogenized using a V-3 Vortex shaker (ELMI, Riga, Latvia) for 3 min at 3000 rpm. A 20.0 µL portion of saliva was taken into a microtube with a dispenser (Eppendorf, Hamburg, Germany), 20.0 µL of concentrated nitric acid was added, and the sample was centrifuged for 10 min at 15,000 rpm using a SM-50 centrifuge (ELMI, Riga, Latvia). Then, the contents of the microtube were transferred into a 10 mL test tube, and 0.2 wt.% nitric acid and 0.1 wt.% Triton X-100 were added to obtain a solution volume of 10.0 mL using a dispenser.

An Agilent 240 FS atomic absorption spectrometer (Agilent, Mulgrave, Australia) was used for determination. Analysis conditions: wavelength, 202.6 nm; sample volume, 10 µL; number of replicate measurements, 3; measurement mode, PROMT Height (5.0%). The temperature program is shown in Table 3.

A calibration function was established with various concentrations of magnesium (0, 10, 25, and 50 µg/L) obtained by automatic dilution from a stock solution (50 µg/L). The prepared samples were randomly divided into 5 series (Table 4). The calibration functions were built after every 12 samples during a daily analysis. Graphite cuvettes have been used for approximately 300 firings without a loss of sensitivity (decrease in absorbance of standard solutions) or degradation of the cuvette material.

The statistical parameters of the analytical method were determined by analyzing the blank and test solutions. Following the recommendations of the International Union of Pure and Applied Chemistry, the parameters involved the limit of quantification (LOQ), linear range, and precision (reproducibility). The LOQ was calculated as 10 *s*/*m*, where *s* is the standard deviation of 10 consecutive blank results and *m* is the slope of the calibration curve. The control sample was a 0.2 wt.% solution of HNO_3_. The linear range was assessed by calculating the linear regression coefficient *R*^2^. The linearity was considered acceptable at *R*^2^ > 0.995. For all linear functions, the linearity range was limited from above by a magnesium concentration of 50 μg/L.

Reproducibility was calculated using the relative standard deviation for 10 consecutive determinations of the selected samples from each series. Recovery (%) was calculated using the addition method: 100 *c*/*c*_ad_, where *c* is the found concentration of the element; the range of values from 90 to 110% was considered acceptable.

### 2.8. Statistical Analysis

STATISTICA 10 software (StatSoft, Tulsa, OK, USA) was used for the data analysis. The Shapiro–Wilk test was used to check the normality of a distribution (*p* ≤ 0.05).

One-way and repeated-measures ANOVAs with Bonferroni correction were used to determine the level and block the effects of behavioral results with the following post hoc analysis with Bonferroni correction.

The Mann–Whitney U test was used to calculate group differences of the behavioral and saliva results. The Wilcoxon matched pair test was used to compare dependent variables (Table 5). A correlation analysis between behavioral and saliva results was computed with the Spearman correlation across all subjects and separately for each group of subjects. The correlations were considered significant if *p* < 0.05.

## 3. Results

### 3.1. Behavioral Results

**Figure 5:**
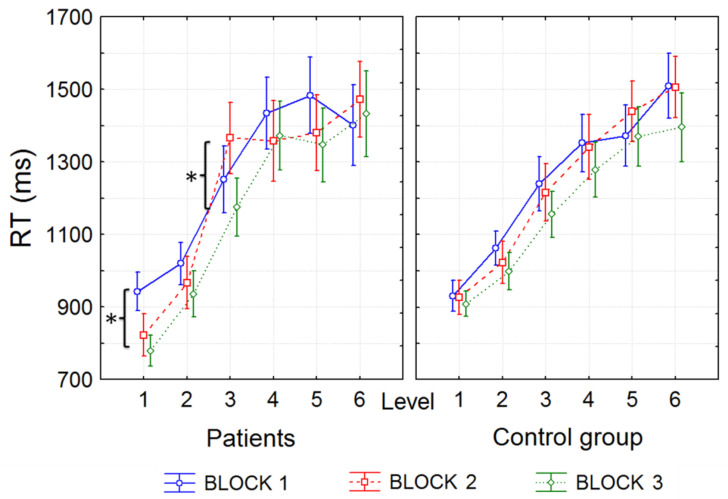
Average indicators of problem-solving time in anxious patients and the control group depending on the level and block; “*” denotes significant differences between the blocks of the same level in the GAD patients; RT is the reaction time.

The reaction time increased from the 1st to 6th level both for GAD patients and healthy volunteers (*F*(5, 130) = 60,016, *p* < 0.001). However, the differences between blocks were significant only in the GAD group (Figure 4). In particular, the reaction time during the 1st level decreased significantly in patients from the first to third block (*p* < 0.05, probabilities for post hoc tests, Bonferroni test), and was significantly lower compared to the control group (Mann–Whitney U test: *z* = −2.3, *p* = 0.03). During the 3rd level of the second block, when the participants received an incorrect negative response (unhappy emoticon), the reaction time significantly increased compared to the other blocks only in anxious patients (*p* < 0.05, probabilities for post hoc tests, Bonferroni test; Figure 5). This section may be divided by subheadings. It should provide a concise and precise description of the experimental results, their interpretation, as well as the experimental conclusions that can be drawn.

The number of correct answers did not differ between the groups of subjects, both on average and separately by levels and blocks. The amount of winnings also did not differ significantly between the groups.

### 3.2. Total Antioxidant Capacity

Since the distribution of the antioxidant capacity values was not normal, the data are presented as median and quartiles Me(Q1; Q3) (Table 6).

No significant differences were obtained at the start point between the groups of controls and GAD patients (the Mann–Whitney test, *p* = 0.235), although the median in the GAD patients was slightly lower. At the endpoint, there were also no significant differences between the groups (the Mann–Whitney test, *p* = 0.463). When comparing data at the beginning and end of the experiment, there were no significant differences for the control group (the Wilcoxon matched pair test, *p* = 0.248), although there was a trend towards a decrease in antioxidant capacity. No significant differences were obtained for the GAD group (the Wilcoxon matched pair test, *p* = 0.465). Thus, we did not obtain significant differences between the groups and time points, which may be due to the small number of subjects in the group. Nevertheless, the high stability of the antioxidant capacity in the GAD during the experiment draws attention.

### 3.3. Magnesium Quantitaion

For all samples analyzed, the relative standard deviation (%) was always below 5%. Since the distribution of magnesium concentrations was not normal, the data are presented as median and quartiles Me(Q1; Q3) (Table 7).

No significant gender differences were found.

Significant differences were obtained at the start point between the groups of controls and GAD patients (the Mann–Whitney test, *p* = 0.033). At the endpoint, there were no significant differences between the groups (the Mann–Whitney test, *p* = 0.272). When comparing data at the beginning and end of the experiment, there were no significant differences for the control group (the Wilcoxon matched pair test, *p* = 0.888). Significant differences were obtained for the GAD group (the Wilcoxon matched pair test, *p* = 0.042). Thus, the level of magnesium in the saliva of anxious patients was initially higher but decreased as a result of problem solving. The magnesium level in the control group was stable during the experiment.

No correlations were found between magnesium and antioxidant capacity.

### 3.4. Correlation Analysis between Saliva and Behavioral Results

A correlation between changes in the antioxidant capacity of saliva and indicators of the success of problem solving was found only in patients with anxiety disorder. In healthy volunteers, no significant correlations were found even at the trend level (*p* > 0.4). There was also no correlation between behavioral results and magnesium content.

The decrease in antioxidant capacity significantly correlated with the time to solve tasks in the second block (levels 3 and 4) in patients with anxiety disorder, when the subjects began to be given incorrect feedback (*r* = 0.84, *p* < 0.001; *r* = 0.86, *p* < 0.001). A similar correlation was observed at the very beginning of problem solving at the first level of the first block. Only anxious subjects showed a significant correlation between the level of oxidative stress and time to solve problems (*r* = 0.74, *p* < 0.001). Moreover, an increase in oxidative stress (a decrease in antioxidant capacity) also correlated with the number of correct answers (*r* = 0.89, *p* < 0.001) and the total gain (*r* = 0.87, *p* < 0.001) only in patients with anxiety disorder.

Thus, even though the performance indicators for solving problems did not differ across the groups (except for the time for solving problems of the first level of the third block), significant efforts to complete the tasks in a group of patients were accompanied by oxidative stress.

## 4. Discussion

Here, we used an anxiety model of problem solving with increasing complexity in GAD patients and healthy volunteers. Incorrect feedback was used as a frustrating factor for an additional psycho-emotional load. Behavioral parameters and changes in the antioxidant potential of saliva and salivary magnesium were studied in comparison with the healthy control group. The main results can be formulated as follows.

1. There was no difference in the results of the quest (the number of correct answers and the total reward); however, anxious participants spent more time (the reaction time) at the moment of experimental frustration due to incorrect feedback and additional negative psycho-emotional load.
2. There were no significant differences in antioxidant capacity in the groups both before and after the experimental session. Average antioxidant capacity also did not change significantly at the endpoint of the experiment. However, the endpoint antioxidant capacity correlated negatively with the reaction time in anxious patients in the second block (where the frustrating factor appeared).
3. Magnesium in saliva was initially significantly higher in the group of anxious participants and decreased at the experiment endpoint; in healthy patients, there were no changes in salivary magnesium at the endpoint.

GAD is associated with autonomic nervous system abnormalities. Hormonally, stress and anxiety have been associated with an increased output of hormones of the hypothalamic–pituitary–adrenal (HPA) axis. However, the vulnerability of these systems in GAD is different. Under psychological stress (public speaking), salivary cortisol did not change, but salivary alpha-amylase (a marker of activation of the autonomic nervous system) sharply increased [52]. During psychological stress tasks, patients with generalized anxiety disorder showed a narrower range in heart rate than controls, which suggested sympathetic inhibition [53]. The possible suppression of adrenergic sympathetic stress responses in GAD is demonstrated elsewhere [54]. GAD patients displayed enhanced autonomic-nervous system (but not the HPA axis) activity vs. healthy controls [55]. As for GAD children, the authors demonstrated a blunted HPA and sympathetic response to acute stress [56]. Thus, the autonomic nervous system is significantly more vulnerable than HPA in generalized anxiety disorder.

The antioxidant capacity of saliva is determined mainly by uric acid, as shown by our studies and other works [36]. The stability of the antioxidant potential of saliva in the anxiety model and the absence of a difference between the groups indicate the stability of this part of the antioxidant network, which confirms the previous studies [17]. The authors noted that in patients with GAD, elevated levels of uric acid may serve as protection against protein oxidation and the formation of aldehydes [18]. HPA stability also helps maintain a stable oxidative state. However, when an additional frustrating factor was added (the incorrect feedback), the reaction time increased and the antioxidant capacity proportionally decreased, which may indicate the consumption of uric acid and the development of oxidative stress. Indeed, psycho-emotional stress is associated with oxidative disbalance [57]. Here, we can conclude that the compensatory possibilities of the metabolism in GAD patients begin to be used up with additional psycho-emotional stress, in contrast to healthy individuals. Thus, patients with GAD in the normal state are compensated in terms of non-lipid oxidative stress but are more vulnerable to additional psycho-emotional stress.

Data on the concentration of magnesium in saliva in healthy individuals differ significantly (see Table 1). Our results are consistent with most published data.

Magnesium is a difficult laboratory marker to interpret. This is due to the fact that ionized serum magnesium makes up a small part of total magnesium, and magnesium, for example, constantly fluctuates inside red blood cells [58]. That is why there are not many studies on magnesium homeostasis, but there are significantly more articles on the effectiveness of nutritional supplements containing magnesium. As an example, a systematic review examines the available evidence for the efficacy of Mg supplementation in the alleviation of subjective measures of anxiety and stress [59].

We found no gender-related differences, which is in line with the literature [60]. Magnesium in saliva is a rather stable analyte [61]. For the laboratory determination of magnesium, saliva is a promising biological sample [62]. Perhaps salivary magnesium will provide new biological information about its homeostasis, while we rely on the data obtained from the analysis of magnesium in the blood and urine. Magnesium is primarily an intracellular cation, and only 2% of magnesium is found in the blood plasma, half of which is free (ionized magnesium), and the remaining half is associated with anions and proteins [63]. Much less similar research has been conducted on saliva, but it can be assumed that magnesium is also present as free and bound species. We centrifuged saliva samples and analyzed only the liquid part that did not contain cells, determining their total magnesium (total Mg). Previous studies suggest that magnesium in saliva and plasma correlate with each other [64]. Thus, by analyzing saliva we can obtain information on systemic homeostasis. This is important in situations where it is necessary to completely eliminate the psycho-emotional stress caused by the sampling of biomaterial.

Magnesium plays an important role in the normal and injured nervous system [21]. The role of magnesium in the biophysics and biochemistry of the nerve impulse is complex and includes both direct and indirect effects [65]. It is known that hyperexcitability of the nervous system is associated with magnesium deficiency [65]. On the other hand, increased activity of the sympathetic nervous system in a stressful situation leads to a decrease in intracellular magnesium caused by noradrenaline [66]. As discussed above, anxiety is associated with autonomic nervous system abnormalities, and therefore magnesium plays a key role. There is a relationship between the disbalance in Mg^2+^ homeostasis and pathological anxiety [67]. However, information on serum magnesium in anxiety disorders is controversial. It has previously been shown that there was no difference in serum magnesium and calcium concentrations in patients with generalized anxiety depression and the healthy controls [22]. However, in anxiety, the partial magnesium reduction took place, associated with a urinary magnesium excretion increase [68]. Magnesium deficiency enhanced anxiety in mice [69].

In sum, magnesium may be redistributed between the intracellular and extracellular pools in chronic anxiety. In patients with GAD, as a hypothesis, under the influence of norepinephrine, magnesium passes from the intracellular to the extracellular space; thus, intracellular hypomagnesemia is possible in these patients. Similar effects were described in patients with chronic heart failure [70]. On the other hand, the high level of magnesium in GAD may be a compensatory mechanism to cope with a decrease in magnesium during anxiety attacks. In acute psycho-emotional stress, healthy individuals possess the balanced stress response system, and changes in magnesium homeostasis are not significant. With acute stress in patients with GAD, the magnesium content decreases, possibly due to increased urinary excretion [68]. This may be due to vasopressin, which is involved in the regulation of anxiety reactions [71,72,73]. Patients with anxiety disorders initially have no abnormalities in the vasopressin concentration [74]. It is possible that under acute stress these patients may experience a decrease in vasopressin as an adaptation of HPA to repetitive stress [75,76]. Decreased vasopressin may be associated with increased magnesium excretion [77]. However, the compensatory capacity of magnesium metabolism in our patients with GAD was sufficient to avoid depletion under the experimental frustrating situation. This mechanism needs to be studied further.

In anxious patients, malondialdehyde had a positive correlation between the parameters of oxidative stress with ionized Mg [78]. In a recent study, the authors studied a lipid oxidative stress marker (malonic dialdehyde) and showed an increase in oxidative stress and magnesium in patients with anxiety. Thus, ionized Mg and indices of oxidative stress could be potential biomarkers in anxious and depressive patients. This is consistent with our findings that GAD patients had elevated magnesium levels. Interestingly, magnesium levels were reduced in the anxiety model, which is also consistent with evidence that magnesium deficiency accompanies anxiety. We did not study lipid oxidative stress, but only the water-soluble part, which is significantly more stable to oxidative load. However, even the urate part began to be depleted in a high psycho-emotional load.

Overall, magnesium metabolism appears to play a key role in the biochemical mechanisms of GAD, and it has a relationship with the oxidative metabolism. Salivary magnesium and antioxidant parameters could give useful information in studying the pathogenesis of GAD and assessing the patients’ condition.

## 5. Conclusions

We used an anxiety model of problem solving with increasing complexity and in-correct feedback as a frustrating factor to assess salivary magnesium and urate antioxidant capacity in GAD patients and healthy volunteers. Compared with the control group, patients with GAD spent more time to solve the task and were more vulnerable to the frustrating factor. The concentration of magnesium in saliva in patients with GAD was higher than in the control group, which can be explained by the influence of hyperactivity of the sympathetic nervous system and the norepinephrine mediator, which contributes to the redistribution of magnesium from cells to the extracellular pool. With psycho-emotional stress, the magnesium content decreased to the level of values in the control group. Thus, the compensatory potential in GAD patients was sufficient to avoid exhaustion during experimental frustrating exposure. The urate antioxidant capacity in patients with GAD without emotional or physical tension was a stable indicator; however, it decreased with additional stress caused by the frustrating factor, which indicates a greater vulnerability of this metabolic part in GAD. We believe that the assessment of salivary magnesium and antioxidant metabolism has great prospects in studying anxiety disorders. The set of determined metabolites should be expanded towards other microelements and other parts of oxidative balance. We also plan to study a new group of patients with GAD in another model of psycho-emotional stress.

## 6. Limitations

There are some limitations of the study. The number of participants was small (17 healthy participants and 15 patients). To avoid the sample size limitation, we attempted to equalize the target and control groups by their age and gender. Moreover, in our study, we recruited only right-handed participants. We also carefully balanced the group of patients. Despite the fact that we recruited only 15 participants, all of them achieved remission (2–6 months ago) and did not take any medication at the time of the study. In this study, we collected saliva only two times (before and after the experimental session). Maybe if we had been collecting saliva after each block, one could see more peculiar changes in the functioning of the hypothalamic–pituitary–adrenal axis and autonomic nervous system. Finally, we assessed only a fraction of the water-soluble antioxidant capacity, but the total antioxidant set includes lipid antioxidants and antioxidant enzymes as well. In future studies, we plan to add these indicators.

## Figures and Tables

**Table 1:** Concentration of salivary magnesium in healthy people.

Reference	Method	Salivary Mg, mg/L
[38]	Inductively coupled plasma mass spectrometry	8.23 ± 3.78
[39]	Inductively coupled plasma mass spectrometry	6.31 ± 0.59
[40]	Flame atomic absorption spectroscopy	10.24 ± 3.49
[41]	Atomic absorption spectroscopy	0.81 ± 0.001
[42]	Inductively coupled plasma mass spectrometry	1.46 ± 0.83
[43]	Flame atomic absorption spectroscopy	7.3 ± 1.7
[44]	Microwave plasma atomic emission spectroscopy	7.6 ± 0.2
This study	Electrothermal atomization atomic absorption spectroscopy	7.6 ± 2.1

**Table 2:** Age, gender, and psychometric scores in studied cohorts; the data are mean ± standard deviation.

Participants	Age	Gender	TMAS ^1^	ESS ^2^
Control group	30.2 ± 10.1	f = 10; m = 7	11.2 ± 5.8	8.7 ± 3.2
Generalized anxiety disorder	28.1 ± 9.3 *p* = 0.69	f = 9; m = 6	33.7 ± 7.4 *p* < 0.0001	8.2 ± 4.2 *p* = 0.78

^1^ Taylor’s Manifest Anxiety Scale—50 item version. ^2^ The Epworth Sleepiness Scale.

**Table 3:** Temperature program (temperature and duration in seconds of the stage) for atomic absorption analysis including drying, charring, atomization, and cleaning.

Drying (°C)/(s)	Charring (°C)/(s)	Atomization (°C)	Cleaning (°C)
95/40	900/8	2200	2200

**Table 4:** The metrological parameters of magnesium quantitation in the experimental sets.

Experimental Set	Limit of Quantitation, µg/L	Linear Regression	*R* ^2^	Reproducibility, %	Recovery, %
Set #1	2.05	y = 0.0049x + 0.0266	0.9971	5.0	103.16
Set #2	2.05	y = 0.0049x + 0.0275	0.9955	4.5	103.16
Set #3	2.05	y = 0.0049x + 0.0261	0.9975	4.9	103.16
Set #4	2.34	y = 0.0043x + 0.0212	0.9997	3.7	101.48
Set #5	2.34	y = 0.0043x + 0.0235	0.9971	4.2	101.48

**Table 5:** Statistical analysis methods used for the data analyses.

Method	One Independent Variable	Levels	Peculiarities of Samples	*p*-Value	Advantages	Disadvantages
One-way and repeated-measures ANOVA with Bonferroni test	Yes	Two levels and more	Independent	Yes	Results are free from errors of scaling and constant bias	Not applied for intercorrelations
Mann–Whitney U test	Yes	Two levels	Independent	Yes	A simple non-parametric test	Requires a number of critical samplings
Wilcoxon matched pair test	Yes	Two levels	Dependent	Yes	Applies to limited sample size, otherwise z-score can be used	No intrinsic clarity for the spread of shift values
The Spearman’s rank correlation test	Yes	Two levels	Dependent	Yes	The assumption of normal distribution is not required	Possibility of misinterpretation

**Table 6:** Salivary antioxidant capacity in the cohorts.

Cohorts	Timing	Me(Q1; Q3), Min	*p*-Values for GAD vs. Control	*p*-Values for after vs. before
Control group	Before the experimental session	44.3 (35.9; 47.0)		
GAD patients		37.6 (32.2; 38.2)	0.38	
Control group	After the experimental session	34.8 (33.1; 35.8)		0.54
GAD patients		37.6 (35.4; 38.6)	0.75	0.86

**Table 7:** Concentration of salivary magnesium in the cohorts.

Cohorts	Timing	Me(Q1; Q3), mg/L	*p*-Values for GAD vs. Control	*p*-Values for after vs. before
Control group	Before the experimental session	7.4 (5.3; 7.9)		
GAD patients		13.5 (11.2; 14.7)	0.04	
Control group	After the experimental session	7.4 (5.5; 9.4)		0.88
GAD patients		7.6 (7.4; 8.8)	0.69	0.04

## Data Availability

The data presented in this study are available on request from the corresponding author. The data are not publicly available due to privacy/ethical restrictions.

## References

[1] DeMartini J., Patel G., Fancher T.L. (2019). Generalized Anxiety Disorder. Ann. Intern. Med..

[2] Malik S., Stead T.S., Mangal R., Ganti L. (2022). General Anxiety Disorder in Youth: A National Survey. Health Psychol. Res..

[3] Gottschalk M.G., Domschke K. (2017). Genetics of generalized anxiety disorder and related traits. Dialogues Clin. Neurosci..

[4] Park S.E., Kim Y.H., Yang J.C., Jeong G.W. (2022). Comparative Functional Connectivity of Core Brain Regions between Implicit and Explicit Memory Tasks Underlying Negative Emotion in General Anxiety Disorder. Clin. Psychopharmacol. Neurosci..

[5] Yuan M., Zhu H., Qiu C., Meng Y., Zhang Y., Ren Z., Li Y., Yuan C., Gao M., Lui S. (2018). Altered regional and integrated resting-state brain activity in general social anxiety disorder patients before and after group cognitive behavior therapy. Psychiatry Res. Neuroimaging.

[6] Hernandez E., Lastra S., Urbina M., Carreira I., Lima L. (2002). Serotonin, 5-hydroxyindoleacetic acid and serotonin transporter in blood peripheral lymphocytes of patients with generalized anxiety disorder. Int. Immunopharmacol..

[7] Gerra G., Zaimovic A., Zambelli U., Timpano M., Reali N., Bernasconi S., Brambilla F. (2000). Neuroendocrine responses to psychological stress in adolescents with anxiety disorder. Neuropsychobiology.

[8] Seddon K., Morris K., Bailey J., Potokar J., Rich A., Wilson S., Bettica P., Nutt D.J. (2011). Effects of 7.5% CO_2_ challenge in generalized anxiety disorder. J. Psychopharmacol..

[9] Bandelow B., Baldwin D., Abelli M., Bolea-Alamanac B., Bourin M., Chamberlain S.R., Cinosi E., Davies S., Domschke K., Fineberg N. (2017). Biological markers for anxiety disorders, OCD and PTSD: A consensus statement. Part II: Neurochemistry, neurophysiology and neurocognition. World J. Biol. Psychiatry.

[10] Bankier B., Barajas J., Martinez-Rumayor A., Januzzi J.L. (2008). Association between C-reactive protein and generalized anxiety disorder in stable coronary heart disease patients. Eur. Heart J..

[11] Tofani T., Mannelli L., Zanardelli M., Ghelardini C., Pallanti S. (2015). P.1.f.003 an immunologic profile study in drug-näive generalized anxiety non depressed patients: A pilot study. Eur. Neuropsychopharmacol..

[12] Wang X., Lin J., Liu Q., Lv X., Wang G., Wei J., Zhu G., Chen Q., Tian H., Zhang K. (2022). Major depressive disorder comorbid with general anxiety disorder: Associations among neuroticism, adult stress, and the inflammatory index. J. Psychiatr. Res..

[13] Dong Z., Shen X., Hao Y., Li J., Li H., Xu H., Yin L., Kuang W. (2021). Gut Microbiome: A Potential Indicator for Differential Diagnosis of Major Depressive Disorder and General Anxiety Disorder. Front. Psychiatry.

[14] Bulut M., Selek S., Bez Y., Karababa I.F., Kaya M.C., Gunes M., Emhan A., Aksoy N., Sir A. (2013). Reduced PON1 enzymatic activity and increased lipid hydroperoxide levels that point out oxidative stress in generalized anxiety disorder. J. Affect. Disord..

[15] Kaya M.C., Bez Y., Karababa I.F., Emhan A., Aksoy N., Bulut M., Gunes M., Atli A., Selek S. (2013). Decreased serum sulphydryl levels as a sign of increased oxidative stress in generalized anxiety disorder. Psychiatry Investig..

[16] Emhan A., Selek S., Bayazit H., Fatih Karababa I., Kati M., Aksoy N. (2015). Evaluation of oxidative and antioxidative parameters in generalized anxiety disorder. Psychiatry Res..

[17] Ercan A.C., Bahceci B., Polat S., Cenker O.C., Bahceci I., Koroglu A., Sahin K., Hocaoglu C. (2017). Oxidative status and prolidase activities in generalized anxiety disorder. Asian J. Psychiatry.

[18] Maes M., Bonifacio K.L., Morelli N.R., Vargas H.O., Moreira E.G., St Stoyanov D., Barbosa D.S., Carvalho A.F., Nunes S.O.V. (2018). Generalized Anxiety Disorder (GAD) and Comorbid Major Depression with GAD Are Characterized by Enhanced Nitro-oxidative Stress, Increased Lipid Peroxidation, and Lowered Lipid-Associated Antioxidant Defenses. Neurotox. Res..

[19] Roomruangwong C., Simeonova D.S., Stoyanov D.S., Anderson G., Carvalho A., Maes M. (2018). Common Environmental Factors May Underpin the Comorbidity between Generalized Anxiety Disorder and Mood Disorders via Activated Nitro-Oxidative Pathways. Curr. Top. Med. Chem..

[20] Islam M.R., Ahmed M.U., Islam M.S., Sayeed M.S., Sadia F., Chowdhury Z.S., Nahar Z., Hasnat A. (2014). Comparative analysis of serum malondialdehyde, antioxidant vitamins and immunoglobulin levels in patients suffering from generalized anxiety disorder. Drug Res..

[21] Cuciureanu M.D., Vink R., Vink R., Nechifor M. (2011). Magnesium and stress. Magnesium in the Central Nervous System.

[22] Islam M.R., Ahmed M.U., Mitu S.A., Islam M.S., Rahman G.K., Qusar M.M., Hasnat A. (2013). Comparative analysis of serum zinc, copper, manganese, iron, calcium, and magnesium level and complexity of interelement relations in generalized anxiety disorder patients. Biol. Trace Elem. Res..

[23] Oddoux S., Violette P., Cornet J., Akkoyun-Farinez J., Besnier M., Noel A., Rouillon F. (2022). Effect of a Dietary Supplement Combining Bioactive Peptides and Magnesium on Adjustment Disorder with Anxiety: A Clinical Trial in General Practice. Nutrients.

[24] Zhang A., Sun H., Wang X. (2012). Saliva metabolomics opens door to biomarker discovery, disease diagnosis, and treatment. Appl. Biochem. Biotechnol..

[25] Jarai T., Maasz G., Burian A., Bona A., Jambor E., Gerlinger I., Mark L. (2012). Mass spectrometry-based salivary proteomics for the discovery of head and neck squamous cell carcinoma. Pathol. Oncol. Res..

[26] Gardner A., Carpenter G., So P.W. (2020). Salivary Metabolomics: From Diagnostic Biomarker Discovery to Investigating Biological Function. Metabolites.

[27] Hyvarinen E., Savolainen M., Mikkonen J.J.W., Kullaa A.M. (2021). Salivary Metabolomics for Diagnosis and Monitoring Diseases: Challenges and Possibilities. Metabolites.

[28] Baima G., Iaderosa G., Citterio F., Grossi S., Romano F., Berta G.N., Buduneli N., Aimetti M. (2021). Salivary metabolomics for the diagnosis of periodontal diseases: A systematic review with methodological quality assessment. Metabolomics.

[29] Nijakowski K., Gruszczynski D., Kopala D., Surdacka A. (2022). Salivary Metabolomics for Oral Squamous Cell Carcinoma Diagnosis: A Systematic Review. Metabolites.

[30] Panneerselvam K., Ishikawa S., Krishnan R., Sugimoto M. (2022). Salivary Metabolomics for Oral Cancer Detection: A Narrative Review. Metabolites.

[31] Sugimoto M. (2020). Salivary metabolomics for cancer detection. Expert Rev. Proteom..

[32] Sturque J., Berquet A., Loison-Robert L.S., Ahossi V., Zwetyenga N. (2019). Interest of studying the saliva metabolome, transcriptome and microbiome in screening for pancreatic cancer. J. Stomatol. Oral Maxillofac. Surg..

[33] Battino M., Ferreiro M.S., Gallardo I., Newman H.N., Bullon P. (2002). The antioxidant capacity of saliva. J. Clin. Periodontol..

[34] Ialongo C. (2017). Preanalytic of total antioxidant capacity assays performed in serum, plasma, urine and saliva. Clin. Biochem..

[35] Peluso I., Raguzzini A. (2016). Salivary and Urinary Total Antioxidant Capacity as Biomarkers of Oxidative Stress in Humans. Pathol. Res. Int..

[36] Gawron-Skarbek A., Prymont-Przyminska A., Sobczak A., Guligowska A., Kostka T., Nowak D., Szatko F. (2018). A comparison of native and non-urate Total Antioxidant Capacity of fasting plasma and saliva among middle-aged and older subjects. Redox Rep..

[37] Baima G., Iaderosa G., Corana M., Romano F., Citterio F., Giacomino A., Berta G.N., Aimetti M. (2022). Macro and trace elements signature of periodontitis in saliva: A systematic review with quality assessment of ionomics studies. J. Periodontal Res..

[38] Inonu E., Hakki S.S., Kayis S.A., Nielsen F.H. (2020). The Association Between Some Macro and Trace Elements in Saliva and Periodontal Status. Biol. Trace Elem. Res..

[39] Romano F., Castiblanco A., Spadotto F., Di Scipio F., Malandrino M., Berta G.N., Aimetti M. (2020). ICP-Mass-Spectrometry Ionic Profile of Whole Saliva in Patients with Untreated and Treated Periodontitis. Biomedicines.

[40] Abbas A., Fadhil N., Gathwan K., Obadi D., Talal S. (2017). Evaluation of Some Salivary Elements in Chronic Periodontitis Patients. IOSR J. Dent. Med. Sci..

[41] Manea A., Nechifor M. (2014). Research on plasma and saliva levels of some bivalent cations in patients with chronic periodontitis (salivary cations in chronic periodontitis). Med. Surg. J..

[42] Huang Y., Zhu M., Li Z., Sa R., Chu Q., Zhang Q., Zhang H., Tang W., Zhang M., Yin H. (2014). Mass spectrometry-based metabolomic profiling identifies alterations in salivary redox status and fatty acid metabolism in response to inflammation and oxidative stress in periodontal disease. Free. Radic. Biol. Med..

[43] Abid Aun W. Inorganic ions level in saliva of patients with chronic periodontitis & healthy subjects. Proceedings of the First National Conference for Iraqi Dental Colleges.

[44] Zhirkov A.A., Yagov V.V., Antonenko A.A., Korotkov A.S., Zuev B.K. (2020). Determination of the Mineral Composition of Human Saliva by Microplasma Atomic Emission Spectroscopy. J. Anal. Chem..

[45] Johns M.W. (1991). A new method for measuring daytime sleepiness: The Epworth sleepiness scale. Sleep.

[46] Taylor J.A. (1953). A personality scale of manifest anxiety. J. Abnorm. Soc. Psychol..

[47] Doskin V.A., Lavrentieva N.A., Miroshnikov M.P., Sharay V.B. (1973). Test of differentiated self-assessment of the functional state. Quest. Psychol..

[48] Bachurina V., Arsalidou M. (2022). Multiple levels of mental attentional demand modulate peak saccade velocity and blink rate. Heliyon.

[49] Portnova G.V., Liaukovich K.M., Vasil’eva L.N., Alshanslaya E.I. (2022). Vegetative and Behavioral Parameters with Increased Cognitive Load in Healthy Volunteers. Pavlov. J. High. Nervious Act..

[50] Arsalidou M., Pascual-Leone J., Johnson J. (2010). Misleading cues improve developmental assessment of working memory capacity: The color matching tasks. Cogn. Dev..

[51] Alekseev A.V., Proskurnina E.V., Vladimirov Y.A. (2012). Determination of Antioxidants by Sensitized Chemiluminescence Using 2,2′-azo-bis(2-amidinopropane). Mosc. Univ. Chem. Bull..

[52] van Veen J.F., van Vliet I.M., de Rijk R.H., van Pelt J., Mertens B., Fekkes D., Zitman F.G. (2009). Tryptophan depletion affects the autonomic stress response in generalized social anxiety disorder. Psychoneuroendocrinology.

[53] Hoehn-Saric R., McLeod D.R., Zimmerli W.D. (1989). Somatic manifestations in women with generalized anxiety disorder. Psychophysiological responses to psychological stress. Arch. Gen. Psychiatry.

[54] Fisher A.J., Newman M.G. (2013). Heart rate and autonomic response to stress after experimental induction of worry versus relaxation in healthy, high-worry, and generalized anxiety disorder individuals. Biol. Psychol..

[55] Tamura A., Maruyama Y., Ishitobi Y., Kawano A., Ando T., Ikeda R., Inoue A., Imanaga J., Okamoto S., Kanehisa M. (2013). Salivary alpha-amylase and cortisol responsiveness following electrical stimulation stress in patients with the generalized type of social anxiety disorder. Pharmacopsychiatry.

[56] Funke R., Eichler A., Distler J., Golub Y., Kratz O., Moll G.H. (2017). Stress system dysregulation in pediatric generalized anxiety disorder associated with comorbid depression. Stress Health.

[57] Salim S. (2014). Oxidative stress and psychological disorders. Curr. Neuropharmacol..

[58] Millart H., Durlach V., Durlach J. (1995). Red blood cell magnesium concentrations: Analytical problems and significance. Magnes. Res..

[59] Boyle N.B., Lawton C., Dye L. (2017). The Effects of Magnesium Supplementation on Subjective Anxiety and Stress-A Systematic Review. Nutrients.

[60] Cieslak M., Jedrzejewska T., Zgirski A. (1990). Determinations of magnesium, iron and copper in the saliva of healthy subjects. Czas. Stomatol..

[61] Czegeny Z.S., Chicharro J.L., Fernandez P., Gutierrez A., Camara C. (2001). Homogeneity and stability studies on sodium, calcium, magnesium, and manganese in human saliva. Biol. Trace Elem. Res..

[62] Machado A., Maneiras R., Bordalo A.A., Mesquita R.B.R. (2018). Monitoring glucose, calcium, and magnesium levels in saliva as a non-invasive analysis by sequential injection multi-parametric determination. Talanta.

[63] Seo J.W., Park T.J. (2008). Magnesium metabolism. Electrolytes Blood Press..

[64] Gradinaru I., Ghiciuc C.M., Popescu E., Nechifor C., Mandreci I., Nechifor M. (2007). Blood plasma and saliva levels of magnesium and other bivalent cations in patients with parotid gland tumors. Magnes. Res..

[65] Durlach J., Bac P., Bara M., Guiet-Bara A. (2000). Physiopathology of symptomatic and latent forms of central nervous hyperexcitability due to magnesium deficiency: A current general scheme. Magnes. Res..

[66] Omiya K., Akashi Y.J., Yoneyama K., Osada N., Tanabe K., Miyake F. (2009). Heart-Rate response to sympathetic nervous stimulation, exercise, and magnesium concentration in various sleep conditions. Int. J. Sport Nutr. Exerc. Metab..

[67] Murck H. (2002). Magnesium and affective disorders. Nutr. Neurosci..

[68] Grases G., Perez-Castello J.A., Sanchis P., Casero A., Perello J., Isern B., Rigo E., Grases F. (2006). Anxiety and stress among science students. Study of calcium and magnesium alterations. Magnes. Res..

[69] Sartori S.B., Whittle N., Hetzenauer A., Singewald N. (2012). Magnesium deficiency induces anxiety and HPA axis dysregulation: Modulation by therapeutic drug treatment. Neuropharmacology.

[70] Samejima H., Tanabe K., Suzuki N., Omiya K., Murayama M. (1999). Magnesium dynamics and sympathetic nervous system activity in patients with chronic heart failure. Jpn. Circ. J..

[71] Appenrodt E., Schnabel R., Schwarzberg H. (1998). Vasopressin administration modulates anxiety-related behavior in rats. Physiol. Behav..

[72] Bunck M., Czibere L., Horvath C., Graf C., Frank E., Kessler M.S., Murgatroyd C., Muller-Myhsok B., Gonik M., Weber P. (2009). A hypomorphic vasopressin allele prevents anxiety-related behavior. PLoS ONE.

[73] Fodor A., Kovacs K.B., Balazsfi D., Klausz B., Pinter O., Demeter K., Daviu N., Rabasa C., Rotllant D., Nadal R. (2016). Depressive- and anxiety-like behaviors and stress-related neuronal activation in vasopressin-deficient female Brattleboro rats. Physiol. Behav..

[74] Uzun N., Akca O.F., Kilinc I., Balci T. (2022). Oxytocin and Vasopressin Levels and Related Factors in Adolescents with Social Phobia and Other Anxiety Disorders. Clin. Psychopharmacol. Neurosci..

[75] Jeong Y.K., Oh Y.I., Song K.H., Seo K.W. (2020). Evaluation of salivary vasopressin as an acute stress biomarker in healthy dogs with stress due to noise and environmental challenges. BMC Vet. Res..

[76] Sugimoto K., Ohmomo H., Shutoh F., Nogami H., Hisano S. (2015). Presentation of noise during acute restraint stress attenuates expression of immediate early genes and arginine vasopressin in the hypothalamic paraventricular nucleus but not corticosterone secretion in rats. Neurosci. Res..

[77] Bouby N., Trinh-Trang-Tan M.M., Bankir L. (1984). Stimulation of tubular reabsorption of magnesium and calcium by antidiuretic hormone in conscious rats. Study in Brattleboro rats with hereditary hypothalamic diabetes insipidus. Pflügers Arch..

[78] Opankovic A., Milovanovic S., Radosavljevic B., Cavic M., Besu Zizak I., Bukumiric Z., Latas M., Medic B., Vuckovic S., Srebro D. (2022). Correlation of Ionized Magnesium with the Parameters of Oxidative Stress as Potential Biomarkers in Patients with Anxiety and Depression: A Pilot Study. Dose-Response.

